# Hepatocellular carcinoma with a direct right atrial extension in an HCV patient previously treated with direct-acting antiviral therapy: a case report

**DOI:** 10.1186/s43044-019-0012-4

**Published:** 2019-09-13

**Authors:** Mahmoud Abdelnabi, Abdallah Almaghraby, Yehia Saleh, Sherif Abd Elsamad

**Affiliations:** 1 Cardiology and Angiology Unit, Department of Clinical and Experimental Internal Medicine, Medical Research Institute, University of Alexandria, 165 El-Horeya Rd, Al Ibrahimeyah Qebli WA Al Hadrah Bahri, Qesm Bab Sharqi, Alexandria Governorate, 21561 Egypt; 2 Department of Cardiology, Faculty of Medicine, University of Alexandria, Alexandria, Egypt; 3 Michigan State University, East Lansing, MI USA

**Keywords:** Hepatocellular carcinoma, Direct extension, Tumor thrombus, Cirrhosis, Direct-acting antiviral therapy, Carcinogenesis

## Abstract

**Background:**

Hepatocellular carcinoma (HCC) is considered the third-leading cause of cancer-related mortality worldwide. Most cases of HCC are usually associated with liver cirrhosis due to various causes such as alcohol or more commonly viral hepatitis. Usually, patients remain asymptomatic for a long time, and symptoms are usually related to the cirrhosis itself or secondary to tumor extension. Intra-cardiac involvement with HCC rarely develops with a very poor prognosis. The occurrence and recurrence of HCC in cirrhotic patients treated with direct-acting antiviral (DAA) therapy (sofosbuvir) have been discussed in a few trials so far with no valid answer.

**Case presentation:**

We are reporting a case of recurrent HCC with an accidentally discovered direct right atrial extension with tumor thrombus through the inferior vena cava (IVC) in a cirrhotic patient previously treated with DAA. Unfortunately, due to his critical general condition, he died within days.

**Conclusion:**

Cardiac involvement in HCC rarely occurs and usually develops in advanced stages of HCC with very poor reported prognosis. Data regarding the relation between DAA and HCC development is controversial.

## Background

Hepatocellular carcinoma (HCC) is the fifth most common malignancy and the third most common cause of cancer death worldwide. It is often a rapidly progressive tumor with high potential for both direct and distant vascular extension. However, despite the high incidence of venous involvement and proximity to the heart, case reports of intracardiac metastasis are quite rare. Here, we are reporting a case of intracardiac extension of recurrent HCC in a previously treated hepatitis C virus (HCV) with DAA with a brief review of cardiac metastasis of HCC and debatable data related to DAA and occurrence of HCC.

## Case report

**Fig. 1:**
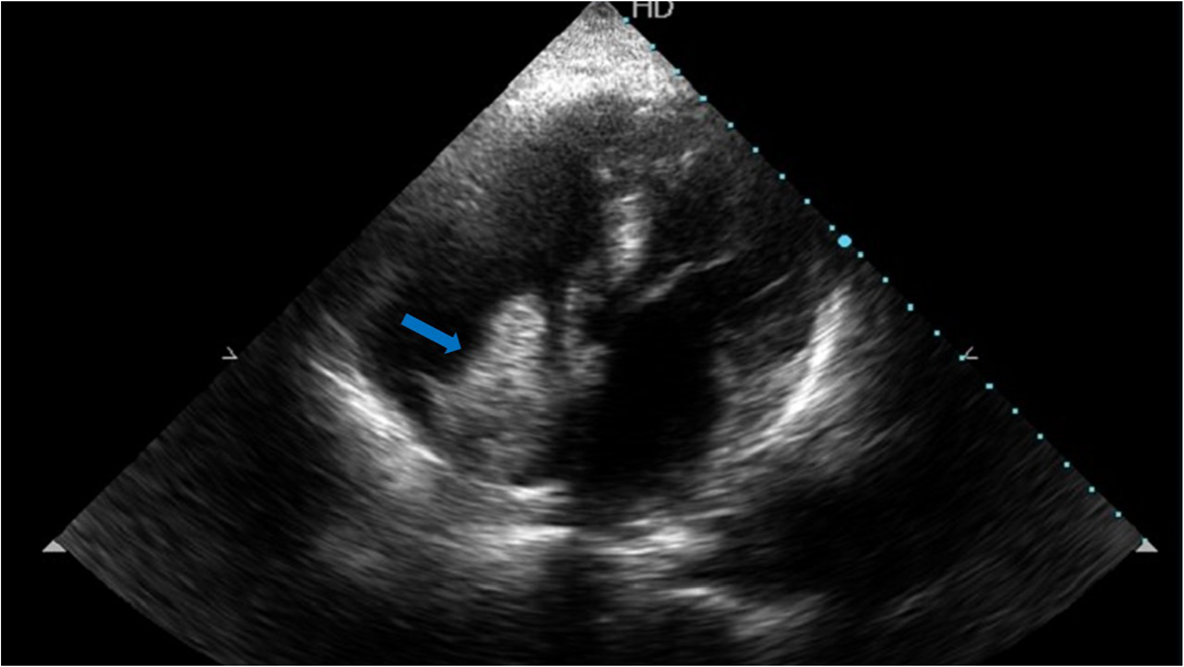
TTE apical four-chamber view showing a large mass in the right atrium marked by a blue arrow

**Fig. 2:**
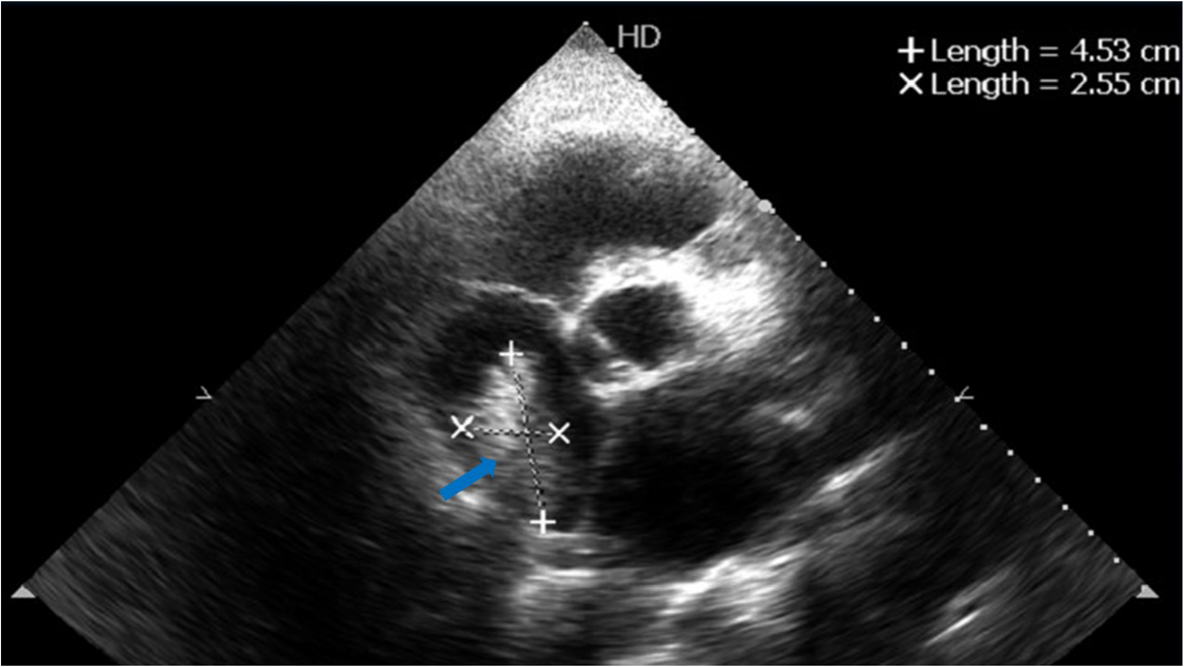
TTE showing a large right atrial mass measuring 4.5 × 2.5 cm marked by a blue arrow

**Fig. 3:**
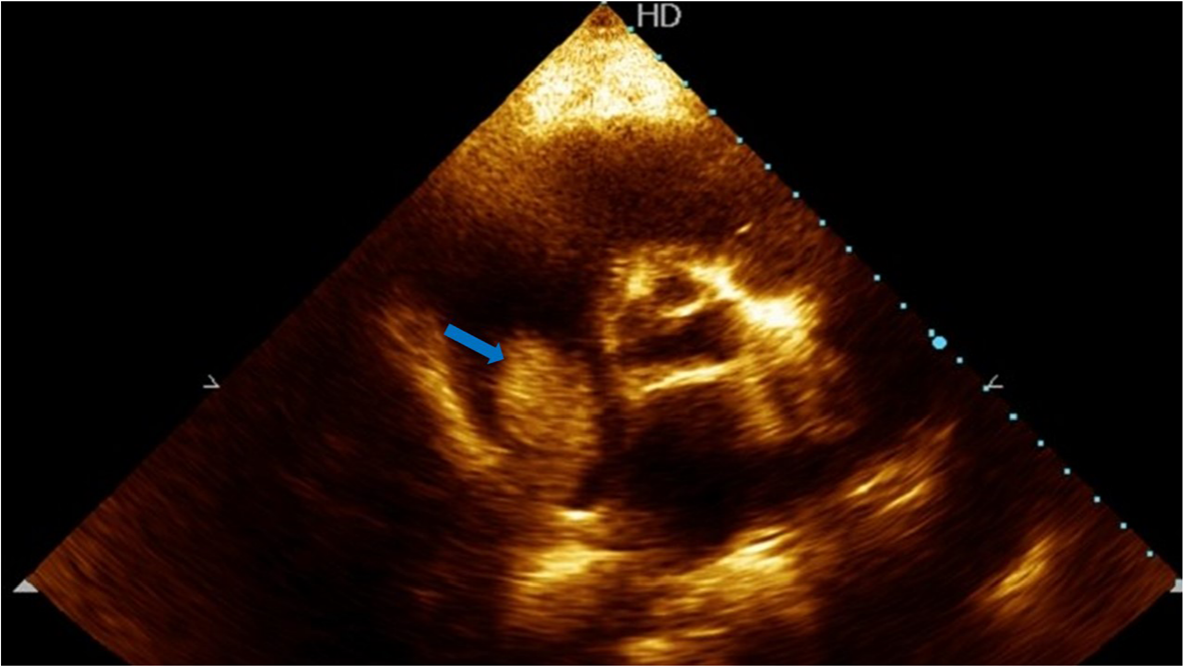
TTE parasternal short-axis view showing a large mass in the right atrium arising from the inferior vena cava marked by a blue arrow

**Fig. 4:**
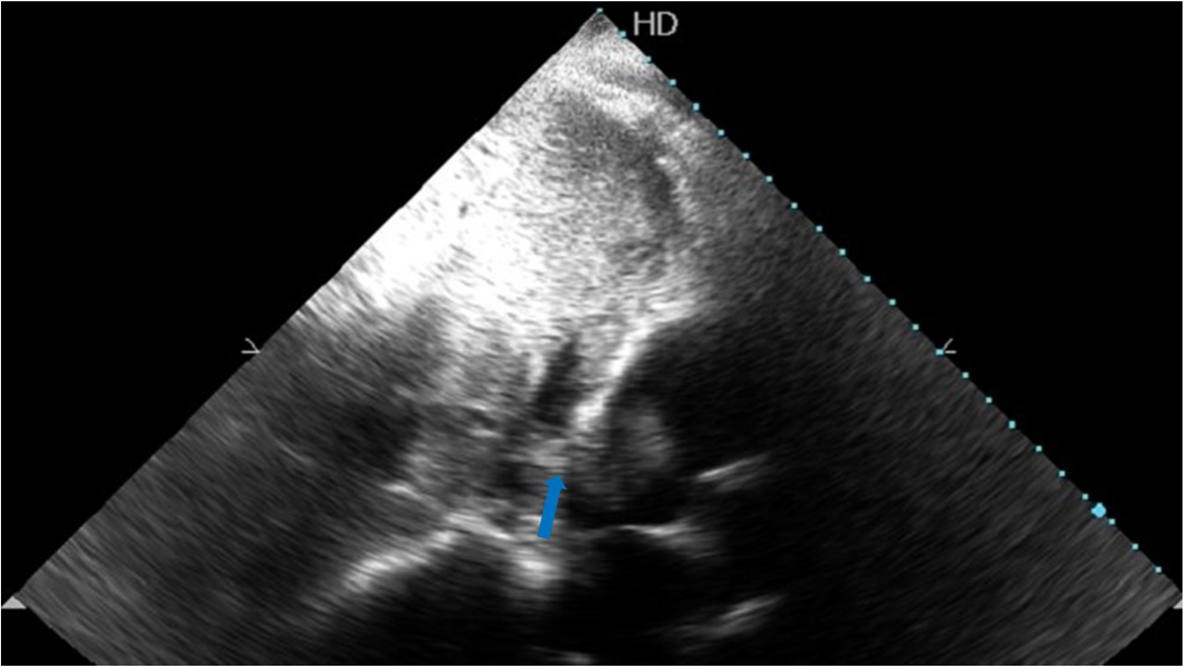
TTE subcostal view showing tumor thrombus extending from the IVC towards the right atrium marked by a blue arrow

A 52-year-old hepatitis C virus (HCV)-positive male is our patient with a known history of transarterial chemoembolization (TACE) for HCC two times, 2 years and one and a half year ago. He was successfully treated with DAA (sofosbuvir) 1 year ago. He presented to our medical facility complaining of deep jaundice, massive ascites, bilateral lower limb edema, and dyspnea. Ultrasonography of the abdomen revealed a mass in the liver extending through the IVC, which is most likely a recurrence of HCC together with a massive amount of ascites. Transthoracic echocardiography (TTE) revealed a highly mobile large cauliflower mass measuring 4.5 × 2.5 cm at the right atrium (Figs. 1 and 2) extending through the IVC (Figs. 3 and 4). Due to patient frailty and hazards of dye in an already renally impaired patient, no further contrast study was done, and only conservative supportive measures were initiated for the management of his deteriorated liver functions, but unfortunately, he passed away after 4 days.

## Discussion

Globally, HCC is considered the fifth most common cancer and the third most frequent cause of cancer-related mortality [1]. More than 80% of HCC cases worldwide are attributed to chronic viral hepatitis B and C infections [2, 3].

HCC is usually asymptomatic until late. Cardiac involvement in HCC rarely occurs and usually develops in advanced stages of HCC. The main mechanism of metastasis into the cardiac cavity is through a direct vascular extension of the tumor to the right side via the hepatic vein and IVC [4].

TTE is a simple readily available noninvasive modality for initial assessment of cardiac involvement in any tumor defining its site, mobility, extension, and effect. Both computed tomography (CT) and magnetic resonance imaging (MRI) can provide further tissue characterization beyond the value of TTE [5]. Cardiopulmonary complications include heart failure, tricuspid stenosis or insufficiency, right ventricular outflow tract obstruction, pulmonary embolism, or even sudden cardiac death [6]. Cardiac surgery and measures to control the growth of HCC have been proposed in young patients with good general condition, yet mostly, the diagnosis is delayed until advanced stages. The reported prognosis of HCC with intra-cardiac involvement is very poor, with a mean survival ranging from 1 to 4 months at the time of diagnosis [7].

There is controversial data regarding the relation between DAA and HCC development. Conte et al. concluded that in patients with HCV-related cirrhosis, DAA-induced resolution of HCV infection was not associated with a reduction of the incidence of HCC occurrence, and previously treated HCC patients have still a high risk of tumor recurrence, in the short term. For these reasons, all cirrhotic patients should be closely monitored and followed during and after antiviral therapy [8]. while Ioannou et al. stated that DAA-induced sustained virologic response was associated with a 71% reduction in HCC risk. Treatment with DAAs was not associated with increased HCC risk compared to interferon (IFN). Further research is required to define whether there is any association between HCC occurrence or recurrence and DAA or it is just related to existing liver cirrhosis.

## Data Availability

The data is available for sharing.

## Abbreviations

CT: Computed tomography
DAA: Direct-acting antiviral
HCC: Hepatocellular carcinoma
HCV: Hepatitis C virus
IFN: Interferon
IVC: Inferior vena cava
MRI: Magnetic resonance imaging
TACE: Transarterial chemoembolization
TTE: Transthoracic echocardiography
